# The U-shaped association between initial serum phosphate level and mortality of traumatic brain injury patients

**DOI:** 10.3389/fneur.2025.1474809

**Published:** 2025-06-18

**Authors:** Ruoran Wang, Xin Yang, Jianguo Xu, Min He

**Affiliations:** ^1^Department of Neurosurgery, West China Hospital, Sichuan University, Chengdu, Sichuan, China; ^2^Operating Room of Anesthesia Surgery Center, West China Hospital/West China School of Nursing, Sichuan University, Chengdu, Sichuan, China; ^3^Department of Critical Care Medicine, West China Hospital, Sichuan University, Chengdu, Sichuan, China

**Keywords:** phosphate, hyperphosphatemia, hypophosphatemia, traumatic brain injury, mortality

## Abstract

**Background:**

Phosphate disorders, including hypophosphatemia and hyperphosphatemia, influence the prognosis of patients. We designed this study to explore the relationship between serum phosphate levels and mortality in traumatic brain injury (TBI) patients.

**Methods:**

TBI patients from the Medical Information Mart for Intensive Care-III (MIMIC-III) database were included in this study. Univariate logistic regression analysis was conducted to identify potential risk factors for TBI mortality. Restricted cubic spline (RCS) analysis was conducted to explore the non-linear relationship between serum phosphate level and TBI mortality, both before and after adjusting for risk factors identified in the univariate logistic regression analysis. Length of ICU stay, length of hospital stay, and 30-day mortality were compared between groups with different serum phosphate levels.

**Results:**

A total of 400 TBI patients died, resulting in an overall mortality rate of 17.6%. Utilizing the RCS analysis, both unadjusted and adjusted associations between serum phosphate levels and TBI mortality were shown as a U-shaped curve. We divided the serum phosphate levels into three groups: < 3.0, 3.0–4.0, and >4.0 mg/dl, according to the U-shaped curve. The multivariate logistic regression analysis revealed that levels < 3.0 mg/dl (*p* = 0.012) and >4.0 mg/dl (*p* = 0.024) were associated with a higher risk of mortality than 3.0–4.0 mg/dl. Mortality rates in these three groups were 19.1%, 14.4%, and 23.4%, respectively (*p* < 0.001). Kaplan–Meier analysis showed a significant difference in survival among the three groups (*p* < 0.001).

**Conclusion:**

Both higher and lower serum phosphate levels are associated with increased mortality in TBI patients. Evaluating serum phosphate levels is beneficial to identify TBI patients at high risk for poor prognosis.

## 1 Introduction

Traumatic brain injury (TBI), the leading cause of long-term disability among adults under 35 years of age, affects an estimated 69 million individuals globally each year (1–4). The 14-day mortality rate among TBI patients undergoing emergency neurosurgery has been reported to be 18% (5). The poor prognosis of TBI is attributable to both initial brain injury severity and complications during hospitalization. As a commonly observed complication among hospitalized patients, electrolyte disorders are particularly prevalent due to pathophysiological changes and a series of iatrogenic interventions. The incidence of electrolyte disturbances in TBI patients ranges from 21% to 82% (6–8). Certain electrolyte disorders, such as hypernatremia, hyponatremia, hyperchloremia, and hypochloremia, have been shown to be associated with increased mortality in TBI patients (9–13).

As an essential trace element, phosphate plays an essential role in maintaining human physiological functions, including cellular signal transduction, energy metabolism, and bone metabolism (14, 15). It is also important to maintain the integrity of the cell membrane and regulate the oxygen release from hemoglobin (14). Although serum phosphate accounts for only 1% of the overall phosphate storage in the human body, changes in the serum phosphate level are commonly observed in various patients and are considered a marker of disease severity. The widely acknowledged normal range of serum phosphate is 2.5–4.5 mg/dl. Serum phosphate levels below or above the normal range are considered hypophosphatemia or hyperphosphatemia. Previous research studies have demonstrated that critically ill TBI patients exhibited lower serum phosphate levels than non-TBI patients, along with a higher prevalence of hypophosphatemia (8, 16). However, to date, no study has explored the incidence of hyperphosphatemia and the effect of phosphate disorders, including hypophosphatemia and hyperphosphatemia, on the prognosis of TBI. Therefore, we designed this study to investigate the incidence of phosphate disorders and verify the prognostic value of abnormal serum phosphate levels in TBI patients.

## 2 Materials and methods

### 2.1 Patients

TBI patients recorded in the Medical Information Mart for Intensive Care-III (MIMIC-III) database were eligible for this study. Developed by researchers at the Massachusetts Institute of Technology (MIT) Laboratory for Computational Physiology (LCP) in collaboration with Beth Israel Deaconess Medical Center (BIDMC), the MIMIC-III database integrates electronic health records, ICU monitoring data, and administrative data of patients hospitalized in the BIDMC between 2001 and 2012. All patients in the MIMIC-III were anonymized and de-identified to protect privacy. To access the MIMIC-III, researchers should complete the “Data or Specimens Only Research” training, sign the data use agreement, agree to ethical use restrictions, and finally download the database from PhysioNet (https://mimic.mit.edu/).

**Figure 1:**
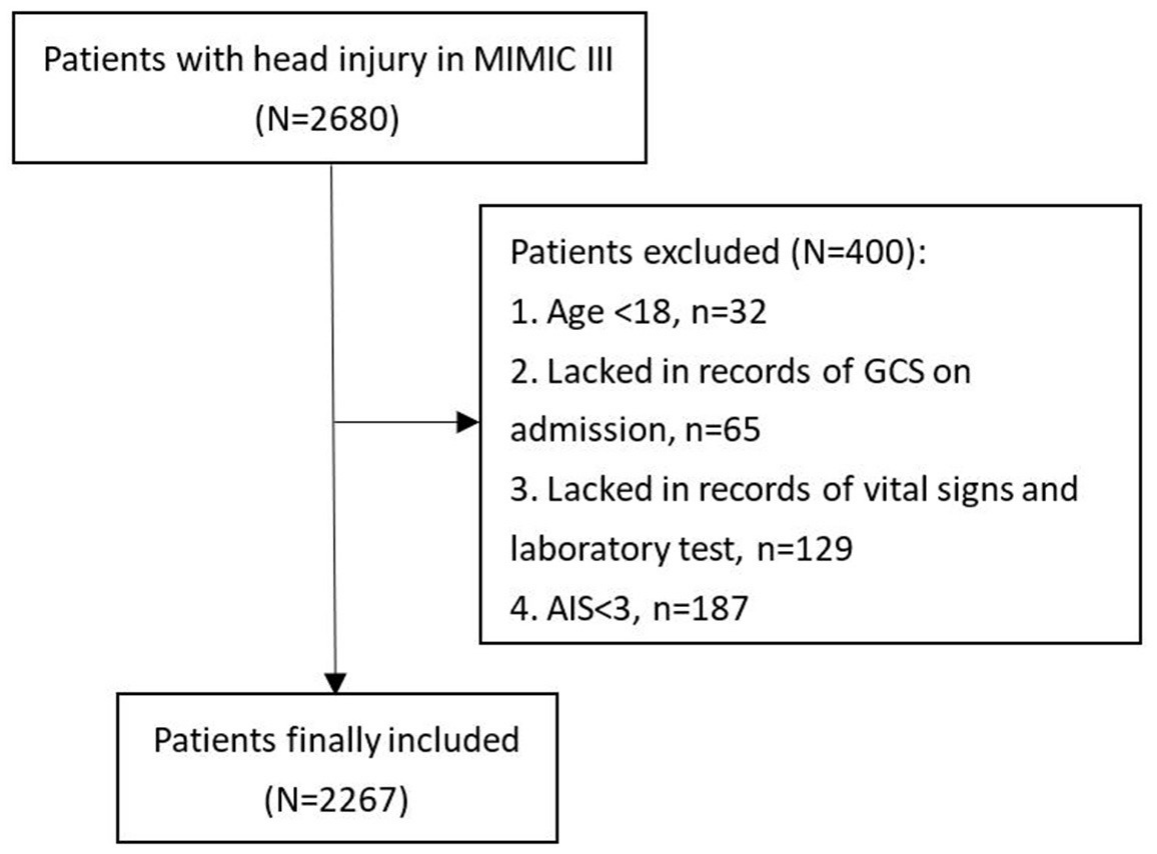
Flowchart of patients' inclusion.

The TBI diagnosis of patients included in our study was identified according to ICD-9 codes (80,000–80,199, 80,300–80,499, and 85,000–85,419). A total of 400 TBI patients were excluded from this study based on the following criteria: (1) age < 18, *n* = 32; (2) lack of records of GCS on admission, *n* = 65; and (3) lack of records of vital signs and laboratory test, *n* = 129; (4) AIS < 3, *n* = 187 (Figure 1). A total of 2,267 patients were finally included in this study. This study was performed according to the ethical standards of the Helsinki Declaration.

### 2.2 Data collection

Baseline data included age, gender, and comorbidities, including diabetes, hypertension, hyperlipidemia, coronary heart disease, history of myocardial infarction, chronic liver disease, chronic renal disease, and cancer. Systolic blood pressure, diastolic blood pressure, pulse oxygen saturation (SpO_2_), Glasgow Coma Scale (GCS), and Injury Severity Score (ISS) were also recorded on admission. Intracranial injury types were classified into epidural hematoma, subdural hematoma, subarachnoid hemorrhage, and intraparenchymal hemorrhage. Laboratory examination results of the first blood sample collected on the first day of hospitalization were analyzed, including white blood cell, platelet, red blood cell (RBC), hemoglobin, blood glucose, serum creatinine, serum calcium, serum phosphate, prothrombin time, and international normalized ratio (INR). Neurosurgical interventions and therapies administered during the first 24 h, including RBC transfusion, platelet transfusion, and vasopressor use, were recorded. The primary outcome was 30-day mortality. The length of ICU stay and length of hospital stay were collected and compared between groups.

### 2.3 Statistical analysis

The Kolmogorov–Smirnov test was utilized to verify the normality of the included variables. Normally distributed or non-normally distributed variables were presented in the form of mean ± standard deviation and median (interquartile range), respectively. The differences in variables with normal distribution or non-normal distribution were assessed using Student's *t*-test and Mann–Whitney *U*-test. The difference in categorical variables was analyzed using the chi-square test or Fisher's exact test. A univariate logistic regression analysis was conducted to identify potential risk factors for TBI mortality. Then, the restricted cubic spline (RCS) was conducted to explore the non-linear relationship between serum phosphate level and TBI mortality, both before and after adjusting for risk factors discovered in the univariate logistic regression analysis. Finally, overall TBI patients were divided into groups according to borderline serum phosphate levels discovered in the RCS curve demonstrating the relationship between serum phosphate level and mortality. Length of ICU stay, length of hospital stay, and 30-day mortality were compared between groups of different serum phosphate levels using the Kruskal–Wallis *H*-test and Fisher's exact test. Additionally, the difference in survival between groups of different serum phosphate levels was compared using the Kaplan–Meier method.

A two-sided *p*-value of < 0.05 was considered statistically significant. Statistical analyses were performed using R (version 3.6.1; R Foundation).

## 3 Results

### 3.1 Baseline characteristics of enrolled TBI patients

Among 2,267 TBI patients selected for the study, 400 died, indicating an overall mortality rate of 17.6% (Table 1). Compared to survivors, non-survivors were older (*p* < 0.001) and had a higher incidence of complications such as diabetes (*p* = 0.002), chronic renal disease (*p* < 0.001), and cancer (*p* = 0.025). Moreover, non-survivors had lower GCS (*p* < 0.001) and higher ISS scores (*p* < 0.001) than survivors. Laboratory examination results revealed that WBC (*p* < 0.001), glucose (*p* < 0.001), serum creatinine (*p* < 0.001), prothrombin time (*p* < 0.001), and INR (*p* < 0.001) were significantly higher in non-survivors, while platelet count (*p* < 0.001), RBC (*p* < 0.001), hemoglobin (*p* < 0.001), and calcium levels (*p* < 0.001) were lower in non-survivors. Importantly, serum phosphate levels did not show a statistical difference between survivors and non-survivors (*p* = 0.715). Finally, compared to survivors, non-survivors had a higher incidence of RBC transfusion (*p* < 0.001), platelet transfusion (*p* < 0.001), vasopressor use (*p* < 0.001), and neurosurgery (*p* = 0.002), as well as a longer ICU stay (*p* < 0.001) but a shorter overall hospital stay (*p* < 0.001).

**Table 1:** Baseline characteristics of included TBI patients.

**Variables**	**Overall patients (*N* = 2,267)**	**Survivors (*N* = 1,867, 82.4%)**	**Non-survivors (*N* = 400 (17.6%)**	***p***
Age (years)	64.9 (43.8–811)	61.3 (41.5–79.4)	77.5 (60.2–85.6)	**<0.001**
Male gender (*n*, %)	1391 (61.4%)	1155 (61.9%)	236 (59.0%)	0.312
**Comorbidities**				
Diabetes (*n*, %)	349 (15.4%)	267 (14.3%)	82 (20.5%)	**0.002**
Hypertension (*n*, %)	844 (37.2%)	685 (36.7%)	159 (39.8%)	0.275
Hyperlipidemia (*n*, %)	298 (13.1%)	243 (13.0%)	55 (13.8%)	0.754
Coronary heart disease (*n*, %)	291 (12.8%)	234 (12.5%)	57 (14.2%)	0.396
History of myocardial infarction (*n*, %)	83 (3.7%)	70 (3.7%)	13 (3.2%)	0.737
Chronic liver disease (*n*, %)	94 (4.1%)	78 (4.2%)	16 (4.0%)	0.981
Chronic renal disease (*n*, %)	153 (6.7%)	103 (5.5%)	50 (12.5%)	**<0.001**
Cancer (*n*, %)	238 (10.5%)	183 (9.8%)	55 (13.8%)	**0.025**
Systolic blood pressure (mmHg)	132 (117–147)	132 (118–147)	132 (113–148)	0.184
Diastolic blood pressure (mmHg)	67 (56–77)	67 (57–78)	66 (53–75)	**0.003**
SpO_2_ (%)	99 (97–100)	99 (97–100)	100 (98–100)	**0.017**
GCS	12 (6–15)	14 (7–15)	6 (3–11)	**<0.001**
ISS	16 (16–25)	16 (16–22)	20 (16–25)	**<0.001**
**Intracranial injury types**				
EDH (*n*, %)	539 (23.8%)	440 (23.6%)	99 (24.8%)	0.660
SDH (*n*, %)	1312 (57.9%)	1080 (57.8%)	232 (58.0%)	1.000
SAH (*n*, %)	949 (41.9%)	769 (41.2%)	180 (45.0%)	0.178
IPH (*n*, %)	447 (19.7%)	377 (20.2%)	70 (17.5%)	0.246
**Laboratory tests**				
WBC (10^9^/L)	11.60 (8.40–15.70)	11.40 (8.30–15.35)	12.80 (9.50–17.10)	**<0.001**
Platelet (10^9^/L)	230 (183–285)	234 (188–288)	213 (162–262)	**<0.001**
RBC (10^9^/L)	4.13 (3.67–4.57)	4.17 (3.73–4.61)	3.88 (3.41–4.36)	**<0.001**
Hemoglobin (g/dL)	12.8 (11.4–14.1)	12.9 (11.6–14.3)	12.0 (10.5–13.4)	**<0.001**
Glucose (mg/dl)	132 (110–165)	127 (107–156)	159 (129–192)	**<0.001**
Serum creatinine (mg/dl)	0.9 (0.7–1.1)	0.9 (0.7–1.1)	1.0 (0.8–1.3)	**<0.001**
Calcium (mg/dl)	8.2 (1.2–8.9)	8.3 (1.2–8.9)	7.9 (1.1–8.8)	**<0.001**
Phosphate (mg/dl)	3.2 (2.8–3.7)	3.2 (2.8–3.7)	3.3 (2.7–3.9)	0.715
Prothrombin time (s)	13.0 (12.3–14.2)	12.9 (12.3–13.9)	13.6 (12.7–16.0)	**<0.001**
INR	1.1 (1.0– 1.3)	1.1 (1.0–1.2)	1.2 (1.1–1.6)	**<0.001**
RBC transfusion during the first 24 h (*n*, %)	176 (7.8%)	116 (6.2%)	60 (15.0%)	**<0.001**
Platelet transfusion during the first 24 h (*n*, %)	223 (9.8%)	155 (8.3%)	68 (17.0%)	**<0.001**
Vasopressor during the first 24 h (*n*, %)	150 (6.6%)	83 (4.4%)	67 (16.8%)	**<0.001**
Neurosurgery (*n*, %)	571 (25.2%)	445 (23.8%)	126 (31.5%)	**0.002**
Length of ICU stay (days)	2.3 (1.2–5.7)	2.1 (1.2–5.1)	3.5 (1.6–6.7)	**<0.001**
Length of hospital stay (days)	6.4 (3.7–12.4)	6.7 (3.8–13.5)	4.8 (2.1–9.1)	**<0.001**

SpO_2_, pulse oxygen saturation; GCS, Glasgow Coma Scale; ISS, Injury Severity Score; EDH, epidural hematoma; SDH, subdural hematoma; SAH, subarachnoid hemorrhage; IPH, intraparenchymal hemorrhage; WBC, white blood cell; RBC, red blood cell; INR, international normalized ratio. Bold values indicated *p* value < 0.05.

### 3.2 Risk factors of mortality among TBI patients analyzed by logistic regression

A univariate logistic regression analysis indicated that age (*p* < 0.001), diabetes (*p* = 0.002), chronic renal disease (*p* < 0.001), cancer (*p* = 0.020), diastolic blood pressure (*p* < 0.001), SpO_2_ (*p* < 0.001), GCS (*p* < 0.001), ISS (*p* < 0.001), WBC (*p* < 0.001), platelet (*p* < 0.001), RBC (*p* < 0.001), hemoglobin (*p* < 0.001), glucose (*p* = 0.008), serum creatinine (*p* < 0.001), calcium (*p* < 0.001), prothrombin time (*p* < 0.001), INR (*p* < 0.001), RBC transfusion (*p* < 0.001), platelet transfusion (*p* = 0.001), and vasopressor use (*p* = 0.002) were potential risk factors of mortality in TBI patients (Table 2).

**Table 2:** Univariate logistic regression analysis of risk factors for mortality in TBI patients.

**Variables**	**OR**	**95% CI**	***p***
Age	1.025	1.019–1.031	**<0.001**
Male gender	1.127	0.905–1.405	0.286
Diabetes	1.545	1.174–2.035	**0.002**
Hypertension	1.138	0.912–1.420	0.251
Hyperlipidemia	1.065	0.778–1.460	0.693
Coronary heart disease	1.160	0.849–1.584	0.352
History of myocardial infarction	0.862	0.472–1.574	0.630
Chronic liver disease	0.956	0.552–1.655	0.871
Chronic renal disease	2.447	1.712–3.495	**<0.001**
Cancer	1.467	1.062–2.026	**0.020**
Systolic blood pressure	0.997	0.993–1.001	0.642
Diastolic blood pressure	0.990	0.983–0.996	**<0.001**
SpO_2_	0.995	0.976–1.015	**<0.001**
GCS	0.821	0.800–0.843	**<0.001**
ISS	1.047	1.035–1.059	**<0.001**
EDH	1.067	0.830–1.371	0.614
SDH	1.006	0.809–1.252	0.955
SAH	1.168	0.940–1.452	0.161
IPH	0.838	0.633–1.111	0.220
WBC	1.038	1.022–1.054	**<0.001**
Platelet	0.997	0.996–0.999	**<0.001**
RBC	0.581	0.498–0.679	**<0.001**
Hemoglobin	0.820	0.779–0.862	**<0.001**
Glucose	1.008	1.006–1.010	**0.008**
Serum creatinine	1.258	1.114–1.420	**<0.001**
Calcium	0.961	0.933–0.989	**<0.001**
Prothrombin time	1.031	1.015–1.048	**<0.001**
INR	1.262	1.108–1.437	**<0.001**
RBC transfusion during the first 24 h	2.664	1.910–3.715	**<0.001**
Platelet transfusion during the first 24 h	2.262	1.662–3.079	**0.001**
Vasopressor during the first 24 h	4.325	3.070–6.091	**0.002**
Neurosurgery	1.469	1.160–1.861	0.251

OR, odds ratio; CI, confidence interval; SpO_2_, pulse oxygen saturation; GCS, Glasgow Coma Scale; ISS, Injury Severity Score; EDH, epidural hematoma; SDH, subdural hematoma; SAH, subarachnoid hemorrhage; IPH, intraparenchymal hemorrhage; WBC, white blood cell; RBC, red blood cell; INR, international normalized ratio. Bold values indicated *p* value < 0.05.

### 3.3 Association between serum phosphate level and TBI mortality

**Figure 2:**
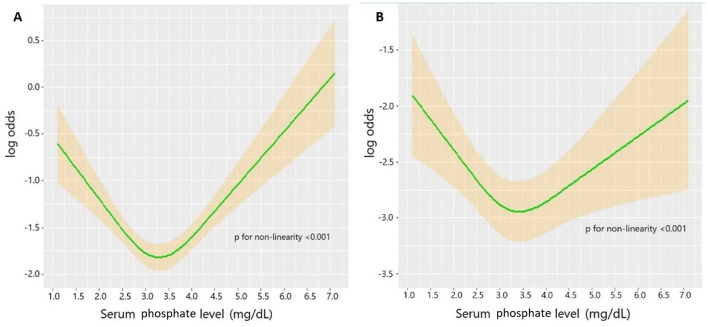
**(A)** Unadjusted association between serum phosphate level and mortality in TBI. **(B)** Adjusted association between phosphate level and mortality in TBI. Adjusted factors include age, diabetes, chronic renal disease, cancer, DBP, GCS, ISS, WBC, platelet, RBC, hemoglobin, glucose, serum creatinine, calcium, PT, INR, vasopressor, RBC transfusion, platelet transfusion, and neurosurgery.

**Figure 3:**
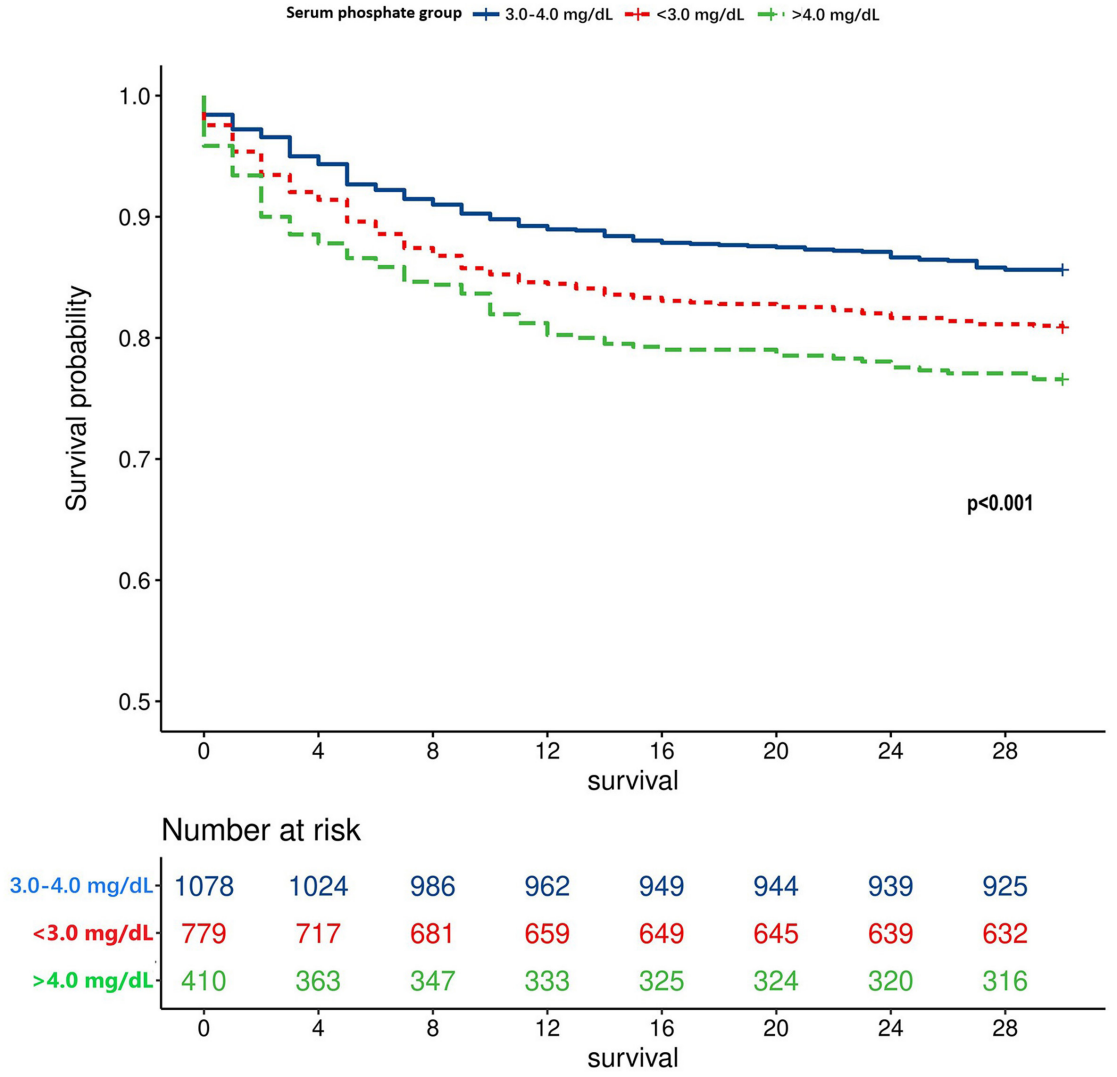
Survival curve of different TBI patients grouped by the serum phosphate level.

Utilizing the RCS, the unadjusted association between the serum phosphate level and the TBI mortality was shown as a U-shaped curve (Figure 2A). After adjusting for potential risk factors in the univariate logistic regression analysis, the adjusted association between the serum phosphate level and TBI mortality remained U-shaped (Figure 2B). Based on the U-shaped curve, we divided the serum phosphate level into three groups: <3.0, 3.0–4.0, and >4.0 mg/dl. The univariate logistic regression analysis indicated that <3.0 mg/dl (*p* = 0.007) and >4.0 mg/dl (*p* < 0.001) were both associated with higher risk of mortality than 3.0–4.0 mg/dl (Table 3). After adjusting for potential risk factors, the multivariate logistic regression analysis indicated that < 3.0 mg/dl (*p* = 0.012) and >4.0 mg/dl (*p* = 0.024) were still associated with a higher risk of mortality than 3.0–4.0 mg/dl. The mortality rate of these three groups was 19.1%, 14.4%, and 23.4%, respectively (*p* < 0.001; Table 4). Length of ICU stay (*p* = 0.077) and length of hospital stay (*p* = 0.720) did not show a significant difference between the three groups. The Kaplan–Meier analysis showed a significant difference in survival between the three groups (*p* < 0.001; Figure 3).

**Table 3:** Multivariate logistic regression analysis of phosphate level and mortality in TBI patients.

**Variables**	**OR**	**95% CI**	***p***
Unadjusted			<0.001
3.0–4.0 mg/dl	1.000	Reference	
< 3.0 mg/dl	1.408	1.101–1.802	0.007
>4.0 mg/dl	1.821	1.369–2.421	<0.001
Adjusted			0.015
3.0–4.0 mg/dl	1.000	Reference	
<3.0 mg/dl	1.454	1.086–1.946	0.012
>4.0 mg/dl	1.510	1.055–2.161	0.024

OR, odds ratio; CI, confidence interval.

Adjusted for age, diabetes, chronic renal disease, cancer, diastolic blood pressure, SpO_2_, GCS, ISS, WBC, platelet, RBC, hemoglobin, glucose, serum creatinine, calcium, prothrombin time, INR, RBC transfusion (*p* < 0.001), platelet transfusion, vasopressor use.

**Table 4:** Mortality comparison between different TBI patients grouped by serum phosphate level.

**Outcomes**	**Overall (*n* = 2267)**	**<3.0 mg/dl (*n* = 779, 34.4%)**	**3.0-4.0 mg/dl (*n* = 1,078, 47.6%)**	**>4.0 mg/dl (*n* = 410, 18.1%)**	***p***
Length of ICU stay (days)	2.3 (1.2–5.7)	2.3 (1.3–5.5)	2.2 (1.1–5.5)	2.6 (1.4–6.3)	0.077
Length of hospital stay (days)	6.4 (3.7–12.4)	6.2 (3.7–12.0)	6.6 (3.7–12.0)	6.5 (3.5–3.8)	0.720
30-day mortality (*n*, %)	400 (17.6%)	149 (19.1%)	155 (14.4%)	96 (23.4%)	<0.001

## 4 Discussion

Phosphate, which is absorbed from the small intestine and excreted by the kidney, plays a crucial role in multiple biological activities, including mineral metabolism, energy production, and cellular signal transduction (17). The reserve of phosphate is regulated by hormones such as 1,25-dihydroxy vitamin D, parathyroid hormone, and fibroblast growth factor 23. Some diseases can disrupt phosphate metabolism and lead to abnormal serum phosphate levels. Typically, serum phosphate levels <2.5 and >4.5 mg/dl are defined as hypophosphatemia and hyperphosphatemia, respectively. The abnormal serum phosphate level is frequently observed among critically ill patients, with the hypophosphatemia prevalence ranging from 10 to 80% and hyperphosphatemia incidence ranging from 9 to 45% (18–20). One previous study showed that the incidence of hypophosphatemia and hyperphosphatemia among TBI patients was 44 and 12%, respectively (8). Multiple trauma patients with TBI had lower serum phosphate levels than those without TBI (8, 16). The phosphate intake amounts were significantly greater in multiple trauma patients with TBI (21).

The abnormal serum phosphate level is attributable to multiple factors, including abnormal absorption, excretion, hormone levels, and disease pathophysiological changes (22). Hypophosphatemia is commonly caused by the transport of inorganic phosphate across cell membranes. The respiratory alkalosis with decreasing blood carbon dioxide level leads to increased intracellular pH, which in turn activates phosphofructokinase to promote glycolysis, incorporating inorganic phosphate transferred from the extracellular fluid (23). Respiratory alkalosis could develop in TBI patients as a result of ventilator-associated hypocapnia due to lower intracranial pressure or unintentional spontaneous hyperventilation (24). Other factors, including refeeding and insulin therapy, could also promote the shift of inorganic phosphate across cell membranes (25–27). Additionally, inadequate ingestion due to dysphagia and gastrointestinal dysfunction, massive loss due to mannitol therapy, excessive catecholamine surge, and increased energy requirements all decrease the serum phosphate level after suffering TBI. Conversely, impaired renal function and hypoparathyroidism led to the accumulation of phosphate in the body with subsequent hyperphosphatemia. Acute kidney injury is prevalent among TBI patients, which may promote the development of hyperphosphatemia.

The influence of hypophosphatemia and hyperphosphatemia on prognosis has been widely verified among various patients. Some studies have shown that hyperphosphatemia or increased serum phosphate level was correlated with higher mortality among patients with acute kidney injury undergoing continuous renal replacement therapy, ST-segment elevation myocardial infarction, blunt trauma, or sepsis (28–33). Hypophosphatemia has also been verified to be associated with poor prognosis of various kinds of diseases, including spontaneous intracerebral hemorrhage and acute kidney injury (34, 35). One previous study found that hypophosphatemia was effective in predicting brain death in severe TBI patients (36). Another confirmed higher serum glucose phosphate ratio reflected more severe injury and poorer prognosis in severe TBI patients (37). Moreover, some studies demonstrated that both hypophosphatemia and hyperphosphatemia were significantly related to the mortality of patients, such as patients with community-acquired pneumonia (38). Our study indicated that abnormally low (<3.0 mg/dl) and high (>4.0 mg/dl) levels of serum phosphate were both significantly correlated with the mortality of TBI patients. The association between the serum phosphate level and TBI mortality is U-shaped, which is similar to findings of previous studies, confirming that serum phosphate level shows a U-shaped relationship with mortality and functional outcome of ischemic stroke patients (39, 40). Although the cutoff values of 3.0 and 4.0 mg/dl do not meet the borderline value of the serum phosphate level defined for hypophosphatemia and hyperphosphatemia, several studies have indicated that even lower or higher phosphate levels within the normal range were associated with higher mortality risk (41, 42).

There are several potential mechanisms to reveal the relationship between phosphate disorder and TBI mortality. Phosphate is an essential component of 2,3-diphosphoglycerate in erythrocytes, which binds with hemoglobin and decreases its affinity between hemoglobin and oxygen, thereby promoting oxyhemoglobin to release oxygen. Therefore, hypophosphatemia would increase the affinity between hemoglobin and oxygen and decrease oxygen release with impaired brain energy metabolism (43). Additionally, the lack of phosphate impairs adenosine triphosphate (ATP) production with the shift from oxidative phosphorylation to glycolysis. The inadequate energy supply leads to the development of organ dysfunction and muscle weakness (44). Conversely, an abrupt increase in serum phosphate may increase reactive oxygen species and decrease nitric oxide by inhibiting endothelial nitric oxide synthase and endothelium-dependent vasodilation (45). Furthermore, hyperphosphatemia has been confirmed, leading to hypocalcemia, cardiac arrhythmia, and cardiac arrest (46).

## 5 Limitations

This study had several limitations. First, all TBI patients included in this study were sourced from a single medical center; thus, generalizability of the findings should be validated in other medical centers in future research. Second, we only analyzed the association between the initial serum phosphate level and the prognosis of TBI but did not record the sequential change in phosphate level and the mean level of serum phosphate during hospitalization. However, the effect of phosphate fluctuation on the prognosis of TBI patients should be explored in the future. Third, although several confounding factors were included, some factors influencing serum phosphate level were not included due to the lack of relevant data in the MIMIC-III, including parathyroid hormone, vitamin D, and phosphate-containing drugs. Finally, although the serum phosphate level was confirmed to be associated with mortality in TBI patients, whether correcting abnormal serum phosphate levels improves the prognosis warrants investigation in future studies.

## 6 Conclusion

Both higher and lower serum phosphate levels are associated with the mortality of TBI patients. Early identification of TBI patients with serum phosphate deviating from the normal value is beneficial to prevent unfavorable prognosis.

## Data Availability

The raw data supporting the conclusions of this article will be made available by the authors, without undue reservation.
